# 'Correction:'Peer chart audits: A tool to meet Accreditation Council on Graduate Medical Education (ACGME) competency in practice-based learning and improvement

**DOI:** 10.1186/1748-5908-2-24

**Published:** 2007-07-27

**Authors:** Lisa J Staton, Suzanne M Kraemer, Sangnya Patel, Gregg M Talente, Carlos A Estrada

**Affiliations:** 1 Department of Internal Medicine, 975 East Third Street Box 94, University of Tennessee College of Medicine-Chattanooga Unit, Chattanooga, TN, USA; 2 Division of General Internal Medicine, Department of Medicine at the Brody School of Medicine at East Carolina University, Greenville, NC, USA; 3 Division of General Internal Medicine, Department of Medicine, University of Alabama at Birmingham, Birmingham, AL, USA

## Abstract

**Background:**

The Accreditation Council on Graduate Medical Education (ACGME) supports chart audit as a method to track competency in Practice-Based Learning and Improvement. We examined whether peer chart audits performed by internal medicine residents were associated with improved documentation of foot care in patients with diabetes mellitus.

**Methods:**

A retrospective electronic chart review was performed on 347 patients with diabetes mellitus cared for by internal medicine residents in a university-based continuity clinic from May 2003 to September 2004. Residents abstracted information pertaining to documentation of foot examinations (neurological, vascular, and skin) from the charts of patients followed by their physician peers. No formal feedback or education was provided.

**Results:**

Significant improvement in the documentation of foot exams was observed over the course of the study. The percentage of patients receiving neurological, vascular, and skin exams increased by 20% (from 13% to 33%) (p = 0.001), 26% (from 45% to 71%) (p < 0.001), and 18% (51%–72%) (p = 0.005), respectively. Similarly, the proportion of patients receiving a well-documented exam which includes all three components – neurological, vascular and skin foot exam – increased over time (6% to 24%, p < 0.001).

**Conclusion:**

Peer chart audits performed by residents in the absence of formal feedback were associated with improved documentation of the foot exam in patients with diabetes mellitus. Although this study suggests that peer chart audits may be an effective tool to improve practice-based learning and documentation of foot care in diabetic patients, evaluating the actual performance of clinical care was beyond the scope of this study and would be better addressed by a randomized controlled trial.

## Background

The Accreditation Council on Graduate Medical Education (ACGME) mandates Practice-Based Learning and Improvement as a core competency area for residents in training. To fulfill this competency, residents are expected to : 1) analyze practice experience and perform Practice-Based Learning and Improvement activities using a systematic methodology, 2) locate appraise and assimilate evidence from scientific studies related to their patients' health problems, 3) obtain and use information about their own population of patients and the larger population from which their patients are drawn, 4) apply knowledge of study designs and statistical methods to appraisal of clinical studies and other information on diagnostics and 5) use information technology to manage information and access on-line information [1]. Continuous Quality Improvement, also called Performance Improvement (PI) projects help to meet this requirement. The improvement activities must relate to the core competencies, involve residents and faculty and produce measurable improvements in patient care or residency education [2].

A chart audit is one quality performance measurement technique which can be used to evaluate residents' competence in Practice-Based Learning and Improvement [3,4]. By itself, chart audit merely measures improvement in performance not competence. A recent pilot study found that self audits led to meaningful physician behavior changes [5], while a Cochrane Collaboration systematic review documented the effectiveness of trained abstractors performing clinical audit with feedback to monitor and improve physician performance [6,7]. While improvements might be due to increased competence in the specific activity of practice-based learning, increased performance could be due to other forms of learning and behaviors as well.

To date there are still few studies evaluating the effectiveness of peer chart audits performed by residents: most studies conducted to date have evaluated self-audits or external audits, and most combined chart audit with formal feedback or an educational intervention [8-11]. Audit-feedback generally involves external audit and relies heavily on the feedback activity for its effectiveness in changing clinical practice. Therefore, the audit-and-feedback strategy fails to recognize that the audit activity itself may have educational value. Little is known about the effectiveness and feasibility of chart audits to meet the ACGME requirements. In addition, the peer chart process itself, in the absence of a formal educational intervention or feedback, has not been studied as a quality improvement technique. We hypothesized that the peer chart audit process itself, without formal educational intervention or feedback, would be associated with improved documentation of foot care.

## Methods

### Setting

The study took place in the three general internal medicine primary care continuity clinics at the Brody School of Medicine at East Carolina University. The Institutional Review Board required written informed consent be obtained from the residents. All patient identifiers were removed at the completion of each audit.

### Participants

Adult patients with diabetes mellitus were identified by searching the electronic medical records (Logician^®^, Medicalogic, GE Medical Systems Information Technologies, Hillsboro, Oregon, USA). Only patients with ICD-9 codes 250.XX in their problem list and receiving continuity care by residents in the categorical and combined internal medicine programs were included.

### Audit Procedures

The chart audits occurred for one-week intervals during continuity clinic conference time. All residents who were present in the clinic during that week participated. Personnel in Medical Records selected the charts of patients who were followed by the residents. The charts were subsequently assigned to the residents. Residents could not audit charts of their own patients, and patient lists were reviewed manually to ascertain that no patient's chart was used more than once per audit.

Audit one was performed in June 2003. Residents were allowed to abstract information dating back for one year prior to June 2003. Audit two occurred in September 2003 for patients seen between July 2003 and September 2003. Audit three was performed in May 2004 for patients seen between October 2003 and May 2004. For audits two and three, the residents were assigned specific visit dates that would encompass visits made after the previous audit to better determine the impact of the audit itself on documenation of care. Charts for repeat audits were selected based on whether the patient had a visit within the time periods above, with no exclusion or inclusion based on whether they had been audited before. No formal feedback was provided to residents between audits. Residents were not informed of the audit until the time of the audit. General Internal Medicine faculty members were aware of the results of the audits, but did not provide formal feedback to residents.

We developed the audit form based on the Diabetes Quality Improvement Project (DQIP) guidelines [12] (see description below) and discussions among general medicine faculty. The form was reviewed and revised for clarity based on consensus, but was not formally piloted. Using the electronic medical record, each resident used the form to review two to five charts during each audit phase. All visits were reviewed to identify the following three domains: (1) history and review of systems, including any mention of the foot or foot problems; (2) foot examination, including performance of the exam and presence of abnormalities; and (3) interventions. An intervention was considered to be present when patients received recommendations for foot care (e.g., prescription for shoes) or were referred for podiatric care or vascular evaluation. The analyses reported here assessed improvements in resident performance related to documentation of the foot examination.

Documentation of the foot exam is described in the Diabetes Quality Improvement Project (DQIP) guidelines [12]. The quality of care standard defined by the DQIP is the percentage of patients receiving a well-documented foot exam. The DQIP foot exam items have been previously validated as predictors for ulceration. The components of a well-documented foot exam include neurological (sensate or vibratory testing with the Semmes-Weinstein monofilament or fork test), vascular (pedal pulses), and skin findings [13].

### Statistical Analyses

Standard descriptive statistics were used and data were analyzed using SPSS^® ^(Chicago, IL). Audits were compared with the chi-square test for trend. The Mantel-Hantzel odds ratio was calculated to quantify the likelihood of interventions between patients with and without abnormalities. The unit of analysis was the patient.

## Results

Residents audited 347 electronic records. Patients had an average of 3.8 (SD 2.5) visits per year during the period of the chart reviews. We observed no increase in documentation of aspects of the history or review of systems related to the feet between audit one (range, 14% to 51%), audit two (range, 15% to 45%) and audit three (range, 11% to 59%) (all p > 0.05). Over time, residents showed improved documentation of the foot exam. Documentation of the neurological exam by the monofilament or fork test (p = 0.001), the vascular exam by assessment of pedal pulses (p < 0.001), and the skin exam (p = 0.005) improved (Figure 1). Documentation of all three exams – neurological, vascular, skin – increased from 6% to 24% (p < 0.001) (Figure 1).

**Figure 1:**
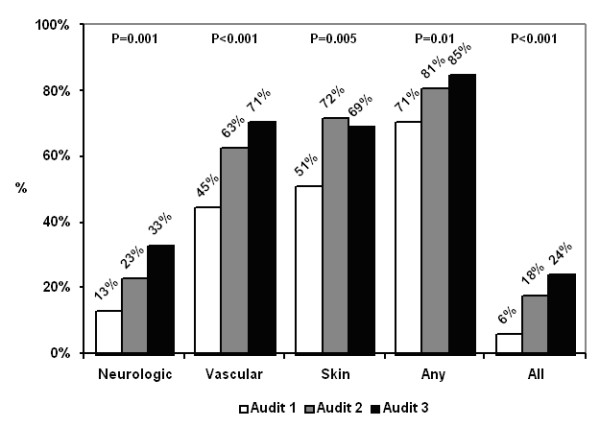
**Foot Exam Documentation**. – *Neurologic *indicates sensate or vibratory testing with the monofilament or fork test at any time, *vascular *indicates pedal pulses evaluation, and *skin *indicates any mention of skin in the feet. *Any *indicates any of the three. *All *indicates all three documented which is a quality of care standard defined by the Diabetes Quality Improvement Program (DQIP): Proportion of patients receiving a well-documented foot exam. P value indicates Chi-Square for trend.

Among audits, we observed no differences in the documented prevalence of foot abnormalities overall, 38% (all p > 0.11), or the frequency of interventions overall, 25% (all p > 0.10). (Table 1). During all three audits, patients with any foot abnormalities received more interventions for foot care as compared to patients without foot abnormalities, [audit one (46% vs. 15%, P = 0.001), audit two (37% vs. 20%, P = 0.02), and audit three (39% vs. 12%, P = 0.002)], data not shown. The odds ratio for any intervention was 3.47 (95% CI 2.09 to 5.75, P < 0.001) for patients with foot abnormalities, as compared to patients without foot abnormalities.

**Table 1:** Diabetic foot documentation

**Variable**	**Total (n = 347)**	**Audit #1 (n = 105)**	**Audit #2 (n = 142)**	**Audit #3 (n = 100)**	**p Value Trend**
Number of visits past year, mean ± SD	3.8 ± 2.5	3.9 ± 2.7	3.4 ± 2.6	3.8 ± 1.9	-
					
**History or Review of Systems**					
Any mention of feet?	170 (51%)	48 (49%)	63 (46%)	59 (59%)	0.16
Any neuropathy symptoms?	107 (32%)	28 (29%)	52 (38%)	27 (27%)	0.80
Any mention of claudication?	47 (14%)	15 (15%)	21 (15%)	11 (11%)	0.38
Any mention of skin problem of feet?	92 (28%)	32 (33%)	36 (27%)	24 (24%)	0.16
Any documented?	189 (55%)	59 (56%)	68 (48%)	62 (62%)	0.42
All documented?	23 (7%)	7 (7%)	11 (8%)	5 (5%)	0.64
					
**Prevalence of Foot Exam Abnormalities**					
Any neurological abnormality?	79 (24%)	19 (19%)	36 (27%)	24 (24%)	0.45
Any vascular abnormality?	54 (16%)	14(14%)	25 (18%)	15 (15%)	0.87
Any skin abnormality?	82 (25%)	27 (28%)	37 (27%)	18 (18%)	0.11
Any abnormality?	132 (38%)	37 (35%)	62 (44%)	33 (33%)	0.76
					
**Intervention for Foot Care**					
Any foot care recommendation?	72 (21%)	19 (20%)	34 (24%)	19 (19%)	0.91
Any foot care referral?	36 (11%)	13 (13%)	17 (12%)	6 (6%)	0.10
Any vascular evaluation referral?	13 (4%)	6 (6%)	3 (2%)	4 (4%)	0.44
Any intervention?	87 (25%)	27 (26%)	39 (28%)	21 (21%)	0.45

## Discussion

This study addressed whether peer chart audit performed by residents, without formal feedback, is associated with improved standards of care for the foot exam in patients with diabetes mellitus. Follow-up chart audit results were associated with a fourfold increase in the number of well-documented foot exams. Although the magnitude of improvement in documentation is statistically significant, the current study was not designed to address what care was actually delivered pre- and post-intervention.

The positive educational impact of the peer chart audits is highlighted by the absence of an extensive instructional component about diabetic foot care. We do not feel that a one-time, half-hour discussion regarding foot care would have had much impact, as past studies with even more extensive physician education have been mixed in terms of demonstrating improved outcomes [14].

The impact of peer involvement may be an important factor contributing to our findings. Studies show that peer coaching, for example, contributes to physicians' professional development of both the learner and the mentor by encouraging reflection time and learning [15]. We suspect that faculty and residents informally engaged in discussions during the process and learned that the foot exam is an important and reliable indicator of care.

We did not see any change in the history or review of systems; other studies have found these items inconsistently asked and documented [16]. This finding may be further explained by the fact that the foot examination is often emphasized as the measure of quality.

Although it is well known that routine visits for patients with diabetes should include advice that they examine their feet daily and obtain an annual foot exam by their provider, studies found that the single most important item of the exam – the neurological exam- was performed in only one third of patients [17,18]. Our findings are consistent with other studies demonstrating less than optimal foot exams and poor adherence to diabetes guidelines [19,20]. For example, in a study by Greenfield et al., the prevalence of foot checks was 61.8% by general internists and 49.6% by endocrinologists [21].

Overall, the data support chart audits as a useful tool for teaching Practice-Based Learning and Improvement. Another study showed that a quality improvement curriculum can produce creative projects that address the core competencies [22]. We also incorporated additional ACGME core competencies including effective patient care, application of medical knowledge to patient care and systems-based practice. In our study we used an accepted standard of care to assess compliance and measure improvement of the foot exam. During the process we learned that implementation was feasible and did not require professional chart abstractors. However, it did require additional personnel, careful planning, and expertise in data management. These additional resources will have financial implications for residency program directors and department heads.

Our study has some limitations. Improvements in foot exam documentation might not reflect changes in practice; we were not able to directly measure practices. Observed improvements might be due to factors other than the peer chart audit activity. For example, the observed changes may have been due to the Hawthorne effect, in which subjects of a study modify their behavior because they are participating in a study [23]. Also, because a variety of other conferences and teaching activities occur elsewhere in our curriculum, it is difficult to control for learning that may have taken place in other forums. However, to our knowledge, no other structured program was implemented at the same time as our chart review. Evidence to more definitively link the peer chart audit activity to observed changes in documentation (and clinical practice) will require a stronger evaluation design such as a randomized controlled trial. Follow-up studies might include a control group of residents, informed of the measurement process but not actually participating in the chart audit process, in order to link the audits to observed improvements.

## Conclusion

A peer chart audit performed by residents, in the absence of formal educational interventions or feedback, was associated with improved documentation of the foot exam in patients with diabetes mellitus. Our conclusions are limited by our study design, and the results observed might be due to other factors rather than the repeated peer reviews. Yet this study demonstrates the feasibility of the peer chart audit method and suggests that an educational tool allowing residents to review the charts of their peers may serve as a reminder of standards of care, and may heighten awareness of the need for quality improvement efforts. The peer chart audit method supports the ACGME recommendations of performance improvement processes by internal medicine residency programs and warrants further evaluation and refinement to support expanded use.

## Competing interests

The author(s) declare that they have no competing interests.

## Authors' contributions

All authors contributed equally to the work. LS conceived the study, participated in the design and coordination and helped draft the manuscript. SK conceived the study and participated in the design and coordination. SP conceived the study and participated in the design and coordination. GT participated in the design and coordination and helped to perform the statistical analysis. CE participated in the design and coordination of the study, helped to draft the manuscript and performed the statistical analysis. All authors read and approved the final manuscript.
